# VEGFA Upregulates FLJ10540 and Modulates Migration and Invasion of Lung Cancer via PI3K/AKT Pathway

**DOI:** 10.1371/journal.pone.0005052

**Published:** 2009-04-01

**Authors:** Chang-Han Chen, Jin-Mei Lai, Teh-Ying Chou, Cheng-Yu Chen, Li-Jen Su, Yuan-Chii Lee, Tai-Shan Cheng, Yi-Ren Hong, Chen-Kung Chou, Jacqueline Whang-Peng, Yu-Chung Wu, Chi-Ying F. Huang

**Affiliations:** 1 Department of Otolaryngology and Kaohsiung Chang Gung Head and Neck Oncology Group, Chang Gung Memorial Hospital-Kaohsiung Medical Center, Chang Gung University College of Medicine, Kaohsiung, Taiwan; 2 Department of Life Science, Fu-Jen Catholic University, Taipei Hsien, Taiwan; 3 Institute of Clinical Medicine, National Yang-Ming University, Taipei, Taiwan; 4 Division of Thoracic Surgery, Department of Surgery, Veterans General Hospital, Taipei, Taiwan; 5 Graduate Institute of Medical Informatics, Taipei Medical University, Taipei, Taiwan; 6 Graduate Institute of Biochemistry, Kaohsiung Medical University, Kaohsiung, Taiwan; 7 Department of Life Science, Chang Gung University, Tao-Yuan, Taiwan; 8 Division of Cancer Center, Wan Fang Hospital, Taipei, Taiwan; Ordway Research Institute, United States of America

## Abstract

**Background:**

Lung adenocarcinoma is the leading cause of cancer-related deaths among both men and women in the world. Despite recent advances in diagnosis and treatment, the mortality rates with an overall 5-year survival of only 15%. This high mortality is probably attributable to early metastasis. Although several well-known markers correlated with poor/metastasis prognosis in lung adenocarcinoma patients by immunohistochemistry was reported, the molecular mechanisms of lung adenocarcinoma development are still not clear. To explore novel molecular markers and their signaling pathways will be crucial for aiding in treatment of lung adenocarcinoma patients.

**Methodology/Principal Findings:**

To identify novel lung adenocarcinoma-associated /metastasis genes and to clarify the underlying molecular mechanisms of these targets in lung cancer progression, we created a bioinformatics scheme consisting of integrating three gene expression profile datasets, including pairwise lung adenocarcinoma, secondary metastatic tumors vs. benign tumors, and a series of invasive cell lines. Among the novel targets identified, FLJ10540 was overexpressed in lung cancer tissues and is associated with cell migration and invasion. Furthermore, we employed two co-expression strategies to identify in which pathway FLJ10540 was involved. Lung adenocarcinoma array profiles and tissue microarray IHC staining data showed that FLJ10540 and VEGF-A, as well as FLJ10540 and phospho-AKT exhibit positive correlations, respectively. Stimulation of lung cancer cells with VEGF-A results in an increase in FLJ10540 protein expression and enhances complex formation with PI3K. Treatment with VEGFR2 and PI3K inhibitors affects cell migration and invasion by activating the PI3K/AKT pathway. Moreover, knockdown of FLJ10540 destabilizes formation of the P110-α/P85-α-(PI3K) complex, further supporting the participation of FLJ10540 in the VEGF-A/PI3K/AKT pathway.

**Conclusions/Significance:**

This finding set the stage for further testing of FLJ10540 as a new therapeutic target for treating lung cancer and may contribute to the development of new therapeutic strategies that are able to block the PI3K/AKT pathway in lung cancer cells.

## Introduction

Lung cancer is the leading cause of cancer-related deaths among both men and women in the world [1]–[2]. Despite recent advances in diagnosis and treatment, the mortality rates remain high, with an overall 5-year survival of only 15%. Surgery remains the first choice of treatment for localized non-small cell lung cancer and provides the best opportunity for cure. However, when first diagnosed, most patients already have advanced disease, and only 35% of patients with non-small cell lung cancer (NSCLC) are eligible for resection [3]. Novel molecular markers or targets aiding in diagnosis and treatment will be crucial for improving the mortality rate.

Tumor invasion and metastasis are important areas for study in order to determine the aggressive phenotype of human cancers and are the major causes of cancer deaths [4]. The process of metastasis is very complex and is considered a late event in tumorigenesis, i.e. cells proliferate, lose contact with neighboring cells, migrate through the interstitial matrix, invade blood and lymph vessels, and deposit into the lymph nodes. Migration and invasion of cells appear to be a result of a complex interplay between the numerous protein families that participate in this process. Mechanisms of cell movement are important not only as part of basic cellular and developmental processes, but also in the pathogenesis of various diseases [5]. To become metastatic, tumor cells must increase the expression of various metastasis-promoting genes. However, in lung cancer, the molecules and mechanisms involved in cell migration or invasion remain largely unknown.

Production and secretion of VEGF-A is commonly observed in most aggressive tumors, and expression of VEGF-A profoundly influences the prognosis of cancer patients, including those with lung cancer [6]–[8]. VEGF-A is one of the most potent stimulators of angiogenesis identified thus far, affecting endothelial cell vascular permeability, proliferation, and motility [7]. Although various intracellular signaling pathways have been proposed to mediate the biological activities of VEGF-A in endothelial cells, the signaling events involved in cell migration and invasion in response to VEGF-A stimulation in lung cancer are not fully understood.

FLJ10540 has several names, including CEP55 [9], C10orf3 [10], and URCC6. CEP55 tagged with GFP-C localizes to the centrosome in interphase cells, to the spindle midzone during anaphase, and to the midbody during cytokinesis [9], [11]–[12]. Furthermore, Cdk1, ERK2, and Plk1 cooperate in the phosphorylation of CEP55 during mitosis, which is required for the correct mitotic localization of CEP55 and its function during cytokinesis [9]. FLJ10540 is overexpressed during human colon [10] and hepatocellular carcinoma [13] tumorigenesis, suggesting that it may function as an oncogene in tumor development. In addition, we previously showed that the overexpression of FLJ10540 contributes to cellular transformation through the activation of PI3K/AKT [13]. However, no large-scale analysis of FLJ10540 expression and its clinicopathologic and functional significance in human lung cancer has been performed.

The aggressive behavior of malignant cancer cells is determined by a complex array of signaling pathways that regulate key functions, such as growth, survival, migration, and invasion. The PI3K/AKT signaling pathway has been causally linked to all four of these responses. [14]–[17]. Further evidence of the importance of PI3K/AKT signaling in cancer comes from studies which have detected overexpression and hyperactivation of PI3K/AKT in a wide range of human tumors, including lung cancer, and this is often linked with poor prognosis [18]. Accumulating evidence from previous reports suggests a potential role of PI3K/AKT in migration and invasion of various cell types, including lung cancer [19], liver cancer [20], breast cancer [21], and pancreatic cancer [22].

In this study, we show that FLJ10540 is overexpressed in lung adenocarcinoma and that ectopic expression of FLJ10540 promotes cell migration and invasion. In human lung adenocarcinoma samples, FLJ10540 positively correlated with VEGF-A expression, as determined by gene expression profiling and IHC staining of tissue microarrays. In addition, stimulation with VEGF-A resulted in an increase in FLJ10540 protein expression in a dose-dependent manner in CL_1-0_ lung cancer cells. Furthermore, FLJ10540 partially translocates from the cytoplasm to the plasma membrane and enhances complex formation with PI3K under VEGF-A stimulation. Finally, VEGF-A affects cell migration and invasion by activating the FLJ10540/PI3K/AKT pathway. These findings suggest that FLJ10540 overexpression is associated with an enhanced metastatic potential and may serve as a potential new therapeutic target for lung adenocarcinoma.

## Results

### Elevated FLJ10540 expression in lung adenocarcinomas and invasive lung cancer cell lines

**Figure 1:**
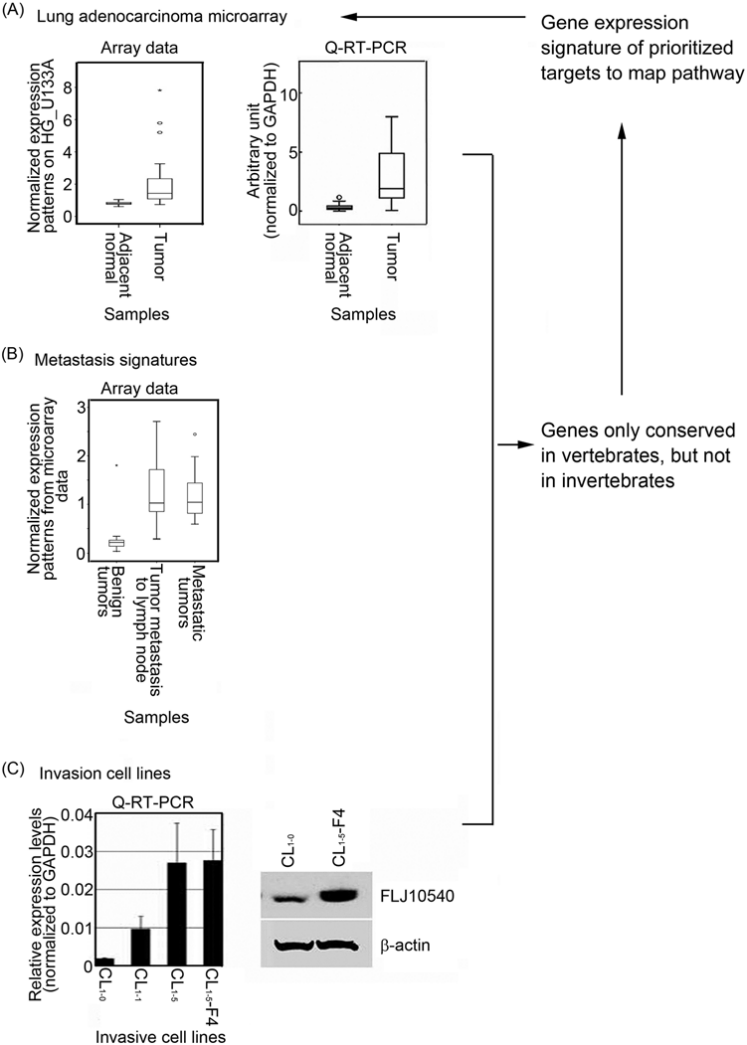
*FLJ10540* exhibits differentially expressed patterns in lung adenocarcinomas, metastatic tumors, and a series of human lung invasive cancer cell lines. (CL_1-0_, CL_1-1_, CL_1-5_, and CL_1-5_-F4, in the order of increasing invasive activity). (A, left panel) The microarray expression patterns of *FLJ10540* in lung adenocarcinoma patients were normalized against the expression patterns on HG_U133A chips. N: adjacent non-tumor tissues; T: tumor tissues. The boxplot shows the data distribution as a grouping classification and indicates that there is a statistically significant difference (*p*<0.0001) between the tumor tissues and the adjacent nont-umor tissues from the same lung cancer patient. (A, right panel) The mRNA levels of *FLJ10540* in samples from 16 lung cancer patients were determined by Q-RT-PCR. The results were normalized against the expression levels of *GAPDH* mRNA in each paired sample and plotted with boxplot. (B) The microarray expression patterns of *FLJ10540* were compared among 30 benign tumors, 20 tumor metastases to the lymph node, and 200 metastatic tumors, and were showed with boxplot. (C, left panel) The mRNA levels of *FLJ10540* were determined by Q-RT-PCR in lung cancer cell lines. The results were normalized against the level of *GAPDH* mRNA in each cell line. The experiments were performed in triplicate. (C, right panel) Total protein was extracted from CL_1-0_ and CL_1-5_-F4 cells and subjected to immunoblot analysis with anti-FLJ10540. β-actin was used an internal loading control.

A significant challenge in the post-genomic era is determining how to prioritize differentially expressed and poorly characterized targets in cancer-related microarray profiling studies. Here, we set up a bioinformatics platform by integrating three different microarray datasets to reveal key regulators involved in the metastasis of lung cancer. Figure 1 shows the schematic data integration approaches and FLJ10540 expression data in different categories. First, samples from 26 patients with lung adenocarcinoma, confirmed by histopathology, were subjected to Affymetrix microarray profiling. Based on the 26 primary lung adenocarcinoma specimens, 826 out of 22,283 differentially expressed transcripts between adjacent non-tumor and tumor areas were clustered by the Wilcoxon signed rank test (*p*<0.01), based on two-fold differences and tested using the Benjamini and Hochberg false discovery rate correlation (*p*<0.01). These molecular portraits, containing 192 up-regulated and 634 down-regulated transcripts, could successfully distinguish the patients' adjacent non-tumor and tumor areas by hierarchical clustering (Figure S1A). Second, public accessible microarray data (downloaded from GEO) were used to acquire different microarray datasets, which were normalized by Quantile Normalization using R to set up potential a metastatic biomarker identification platform. We compared 225 secondary metastatic tumors (including 20 tumor metastases to the lymph node and 200 metastatic tumors) and 30 benign tumors to obtain sets of potential metastatic oncogenes (871) and metastatic suppressor genes (1042). Finally, to prioritize the targets and to validate our metastasis-related genes in cell lines, a series of lung adenocarcinoma cell lines, including CL_1-0_, CL_1-1_, CL_1-5_ and CL_1-5_-F4, with increasing degrees of invasiveness [23], was also subjected to microarray profiling. A total of 6,238 transcripts had higher expression levels in the high invasion ability cell line CL_1-5_-F4 than the parental CL_1-0_ cells. The intersection of these three datasets reveals many novel targets. We characterized the genes that are conserved only in vertebrates, but not in invertebrates, to reveal some novel human cancer-specific abnormalities. As a result, we focused on the poorly characterized gene *FLJ10540*.

*FLJ10540* exhibits up-regulated expression patterns in lung adenocarcinomas (Figure 1A, left panel) and metastatic signatures (Figure 1B). To validate the gene expression profiling data for *FLJ10540*, Q-RT-PCR was performed on 16-pairwise lung adenocarcinoma tumor and adjacent non-tumor samples. Figure S1B shows that overexpression of *FLJ10540* was observable in our lung patient samples, with 15 out of 16 lung patient tumors (94%) showing a higher signal after normalization to *GAPDH* for equal template loading. The average expression level of *FLJ10540* in lung adenocarcinomas was 8 fold higher than that in adjacent non-tumor lung tissue samples, as analyzed by boxplot (Figure 1A, right panel). We next verified the expression of FLJ10540 in representative and freshly frozen lung adenocarcinoma and adjacent non-tumor samples by immunohistochemistry staining using anti-human FLJ10540 antibodies. Our data indicate that the level of FLJ10540 was increased in the tumor samples, as compared to that in the adjacent non-tumor tissues (data not shown).

### Expression of FLJ10540 in a series of invasive human lung adenocarcinoma cancer cell lines

During lung tumor development, one of the most critical transitions is the progression from *in situ* to invasive carcinoma. To explore the possible role of FLJ10540 in the invasiveness of lung adenocarcinoma cells, we first examined the expression of *FLJ10540* in a panel of cell lines, CL_1-0_, CL_1-1_, CL_1-5_, and CL_1-5_-F4, with either low or high invasive abilities, as previously described [23] by Q-RT-PCR. The data indicated that levels of *FLJ10540* mRNA were high in CL_1-5_ and CL_1-5_-F4 cells that have high invasive and metastatic abilities, but were lower in the low invasive CL_1-0_ cells (Figure 1C, left panel). FLJ10540 protein levels were also markedly higher in the highly invasive CL_1-5_ cells than in the low invasive CL_1-0_ cells, as shown by western blot analysis (Figure 1C, right panel). The results indicated that the upregulation of FLJ10540 mRNA and protein expression was positively correlated with the invasive capability of the lung cancer cells, raising the possibility that upregulation of *FLJ10540* might lead to some of the abnormalities found in human lung adenocarcinoma.

### Overexpression of FLJ10540 promotes cell migration and invasion

**Figure 2:**
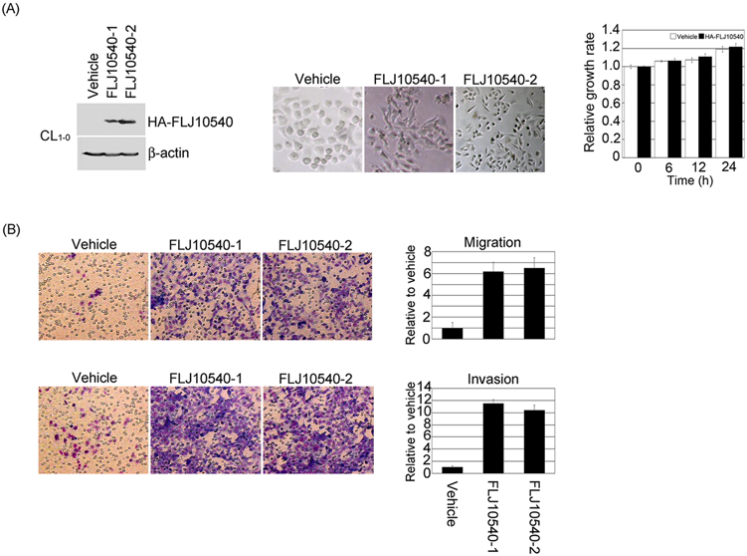
FLJ10540 overexpression promotes lung cancer cell migration and invasion. (A, left panel) HA-tagged-FLJ10540 stable clones of CL_1-0_ cells were established. The cell lysates were subjected to immunoblot analysis with anti-HA antibody. (A, middle panel) Phase-contrast images of monolayer cultures of CL_1-0_ cells expressing FLJ10540 and vehicle control were shown. (A, right panel) To measure cell growth rates, 5×10^3^ cells of vehicle-CL_1-0_ and CL_1-0_-FLJ10540 stable clones were plated at day 0 in 96-well plates with 10% FBS. The cells were counted at indicated time points by the MTT assay (OD_570_) to quantify the cell growth. Data were normalized against the OD_570_ value on day 0. The growth rate of CL_1-0_ cells is shown as the mean±s.d. of three independent experiments. (B, upper panel) For the migration assay, 5×10^3^ cells of vehicle-CL_1-0_ and CL_1-0_-FLJ10540 stable clones were seeded into the top of a Transwell insert. After 24 hours, the cells on the topside were scraped, and the cells that migrated to the bottom were fixed and stained with Giemsa. The migration relative-fold of vehicle-CL_1-0_ and CL_1-0_-FLJ10540 stable clones was normalized with vehicle control and presented diagrammatically. (B, bottom panel) For the invasion assay, 1×10^4^ cells were seeded after Matrigel was added. The invasion relative-fold of stable clone was normalized against vehicle cells and represented diagrammatically. All of the data represent the mean±s.d. of three independent experiments.

Since FLJ10540 is overexpressed in lung adenocarcinoma and a highly invasive lung cancer cell line, it seemed possible that its expression might affect cell motility. To clarify role of FLJ10540 in motility in the mammalian cells, we examined whether overexpression of FLJ10540 was able to affect cell migration and invasion. Two different cell types were employed to evaluate this possibility. First, CL_1-0_ cells stably expressing hemagglutinin (HA)-tagged FLJ10540 were established. Several stable transfectants with increasing levels of the FLJ10540 protein were selected (Figure 2A, left panel) for further studies. Interestingly, FLJ10540 overexpression in CL_1-0_ cells was associated with strikingly altered cell morphology. FLJ10540 overexpressed clones were decreased in cell size and appeared to be more spindle-shaped and had increased intercellular separation compared to vehicle control clones, which were round-shaped (Figure 2A, middle panel). However, the proliferation rate, as determined by the MTT assay, was not significantly different from that of the vehicle control or cells expressing FLJ10540 during 24 hours (Figure 2A, right panel). To examine the effect of FLJ10540 on human lung cancer cell migration, stable cell lines expressing HA-FLJ10540 or vehicle control were seeded on Transwell chambers. The results showed that FLJ10540 CL_1-0_-stable transfectants could increase their migration, as measured by the Transwell migration assay; this was compared to the vehicle control, where very few cells migrated (Figure 2B, left upper panel). Quantitatively speaking, the migration ability of FLJ10540 CL_1-0_-stable transfectants was approximately 6–7 fold higher than that of the vehicle control (Figure 2B, right upper panel). We next carried out an *in vitro* invasion assay using a Matrigel coated barrier filter. After a 24 hour incubation in a Matrigel-coated Transwell chamber, FLJ10540 CL_1-0_-stable transfectants had invaded the basement membrane material and passed through the pores to reach the underside of the filter (Figure 2B, left bottom panel). Similarly, FLJ10540 CL_1-0_ expressing cells showed a 10–12 fold higher invasion ability than the vehicle control cells (Figure 2B, right bottom panel).

Second, to avoid clonal effects, a retroviral-based Flag-tagged FLJ10540 was infected into an RK3E cell line (Figure S2A) that had previously been used successfully to evaluate migration or invasion induced by different genes and signaling pathways, such as fibroblast growth factor 9 (FGF9) [24], k-ras [25], and Snail [26]. RK3E cells exhibit multiple features of epithelia, such as a near-normal karyotype and a low background of cell migration and invasion. Mixed pools of infected RK3E cells were used for the migration and invasion assays. Again, FLJ10540 expressing cells showed a 4–5 fold higher migration ability than the vehicle control cells (Figure S2B) and a 7 fold higher invasion ability than the vehicle control cells (Figure S2C). Taken together, these findings support the hypothesis that FLJ10540 can promote cell migration and invasion in mammalian cells.

### Knock-down of endogenous FLJ10540 by siRNA suppresses lung cancer cell migration and invasion

**Figure 3:**
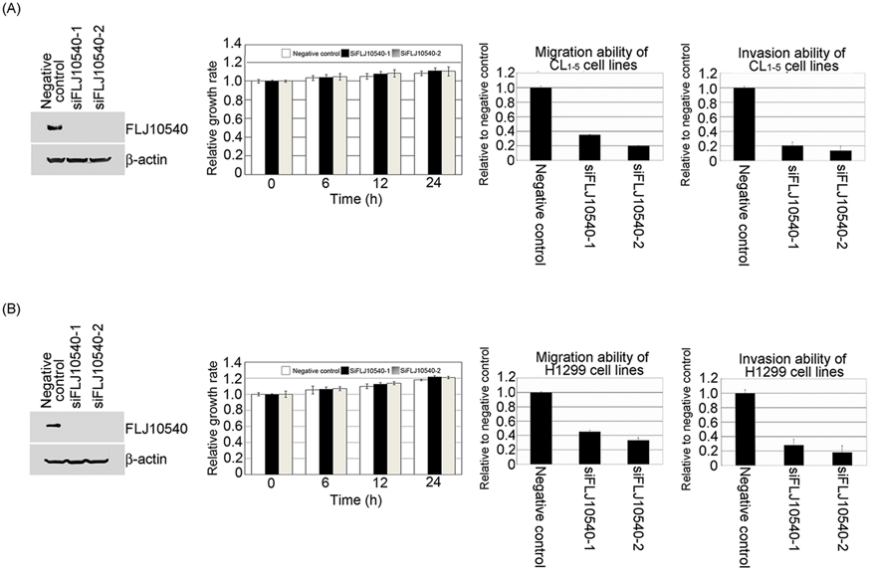
The migration and invasion abilities of lung cancer cells are inhibited by FLJ10540-mediated siRNAs. (A and B, left panel) A negative control siRNA with two different FLJ10540 siRNAs (siFLJ10540-1 and siFLJ10540-2) were transfected into CL_1-5_ and H1299 cells for 24 hours. After transfection, western blotting was performed using anti-FLJ10540 and anti-β-actin antibodies. (A and B, middle panel) To measure cell growth rates, 5×10^3^ cells of negative control-CL_1-5_, CL_1-5_-FLJ10540 siRNAs, negative control-H1299, and H1299-FLJ10540 siRNAs were plated at day 0 in 96 well plates with 10% FBS. The cells were counted at indicated time points by the MTT assay (OD_570_) to quantify the cell growth. Data were normalized against the OD_570_ value on day 0 of each treatment. The growth rate of CL_1-5_ and H1299 cells are shown as the mean±s.d. of three independent experiments. (A and B, right panel) The migration relative-fold and the invasion relative-fold of CL_1-5_ and H1299 were normalized against negative control cells and represented diagrammatically. The results represent the mean±s.d. of three independent experiments.

To confirm that FLJ10540 affects cell migration and invasion in human lung cancer cells, we used a siRNA approach to inhibit endogenous expression of FLJ10540 and then assayed the migration and invasion abilities of CL_1-5_ and H1299 cells. First, to examine the specificity of siRNAs, an HA-tagged FLJ10540 expression construct was co-transfected with three different siRNAs; these consisted of two chemically synthesized siRNAs specific for FLJ10540 (siFLJ10540-1 and siFLJ10540-2) and a negative control siRNA, which were each transfected into HEK293T cells. Both FLJ10540 siRNAs showed almost complete inhibition of HA-tagged FLJ10540 expression, as shown by western blot analysis using an anti-HA antibody, while the level of HA-tagged FLJ10540 remained high with the negative control siRNA (data not shown). Second, to examine if the specific FLJ10540 siRNAs could inhibit endogenous FLJ10540 expression in CL_1-5_ and H1299 cells, we transfected siRNAs into CL_1-5_ and H1299 cells for 24 hours, followed by western blot analysis using an antibody against FLJ10540. The data showed a dramatic reduction in the FLJ10540 protein levels with both FLJ10540 siRNAs, and there was no significant reduction of FLJ10540 when the negative siRNA was used (Figure 3A and B, left panel). However, the proliferation rate, as determined by the MTT assay, was not significantly different between the negative control cells and cells expressing FLJ10540 siRNAs during 24 hours (Figure 3A and B, middle panel). Third, to study the effect of siRNA FLJ10540 transfection on migration, negative control and siRNA FLJ10540-transfected CL_1-5_ or H1299 cells were separately seeded on Transwell chambers with uncoated filters. After 24 hour incubation, the motility potential of siRNA FLJ10540 cells was found to have been strongly inhibited by the siRNAs (Figure 3A and B, right panel). Finally, the same panels of negative or FLJ10540 siRNA transfected cells were seeded in a Matrigel-coated Transwell chamber. After 24 hours of incubation, the invasive potential of the siRNA FLJ10540 cells appeared to be significantly reduced (Figure 3A and B, right panel). These results strongly support the idea that FLJ10540 plays a role in cell migration and invasion in human lung cancer cells.

### Using the concept of syn-expression to uncover VEGF-A, but not VEGF-B, as the upstream regulator of FLJ10540

**Figure 4:**
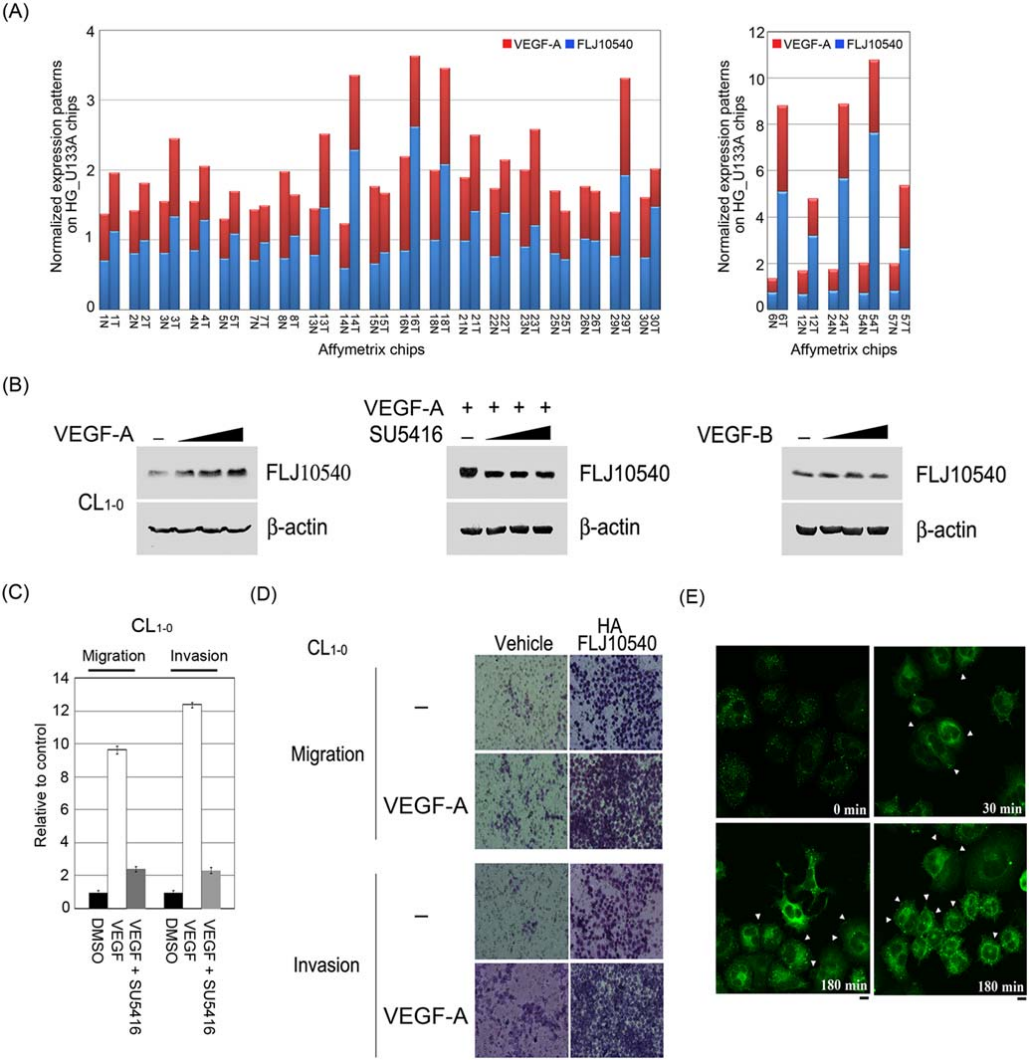
VEGF-A promotes FLJ10540 protein expression and enhances FLJ10540-induced migration and invasion in lung cancer cells. (A) The microarray expression patterns of *VEGF-A* and *FLJ10540* in lung adenocarcinoma patients were shown. The results were normalized against the expression patterns of 56 chips (HG_U133A). N: adjacent non-tumor tissues; T: tumor tissues. (B, left panel) VEGF-A induced an increase in FLJ10540 protein levels in a dose-dependent manner. After treatment with various concentration of VEGF-A (left panel) or VEGF-B (right panel) for 10 min in CL_1-0_ cells, the total proteins were extracted from CL_1-0_ cells and probed with antibody against FLJ10540. (B, middle panel) Serum-starved CL_1-0_ cells were pre-treated with or without various concentrations of SU5416 for 2 hours; cells were then stimulated with 20 ng/ml VEGF-A for 10 min. β-actin was used as an internal loading control. (C) Serum-starved CL_1-0_ cells were pre-treated with or without SU5416 for 2 hours; cells were then stimulated with VEGF-A (at the concentration of 20 ng/ml) for 3 hours. The migration and invasion relative-folds were normalized against vehicle cells. (D, left, upper panel) For the migration assay, 5×10^3^ cells of vehicle-CL_1-0_ and CL_1-0_-FLJ10540 stable clones were seeded into the top of a Transwell insert and allowed to adhere for 12 hours, and were then incubated with or without VEGF-A (20 ng/ml) for 3 hours. At the end of the assay, the cells on the topside were scraped, and the cells that migrated to the bottom were fixed and stained with Giemsa. (D, left, bottom panel) For the invasion assay, 1×10^4^ cells were seeded after Matrigel was added, and were then incubated with or without VEGF-A (20 ng/ml) for 3 hours. All of the data represent the mean±s.d. of three independent experiments. (E) Indirect immunofluorescence analysis of FLJ10540 in VEGF-A-treated cells. The protein expression and the subcellular localization of FLJ10540 were analyzed in the presence or absence of VEGF-A (20 ng/ml) in CL_1-0_ cells using immunofluorescence microscopy. After being incubated with or without VEGF-A for 30 min or 180 min, the cells were fixed with 3.7% formaldehyde and processed for indirect immunofluorescence microscopy. FLJ10540 translocated to the cell membrane upon VEGF-A treatment (Arrowhead). Bar: 10 µm.

Genes belonging to the same pathway often show the same expression profiles, which is referred to as syn-expression [27]. Hence, to map the potential signaling pathway or upstream regulator(s) participating in FLJ10540-elicited migration and invasion characteristics, we employed our lung cancer microarray dataset, as described earlier, to gain insight into the functional concordance of co-expressed genes. To test this hypothesis, we first examined whether the mRNA expression profile of *FLJ10540* correlates with any ligand or receptor in our lung cancer microarray database (Figure 1). Of these co-expressed genes, the top candidate is *VEGF-A*, which was highly positively correlated with the mRNA expression level of *FLJ10540* in paired lung cancer patients (r = 0.7 p<0.001) (Figure 4A). VEGF-A is known to be a critical pro-angiogenic factor, with abilities to regulate many steps in the angiogenic process, including proliferation, migration, invasion, and survival [28], [29]. In fact, VEGF-A is highly expressed in many malignant tumors, including lung cancer [30], [31], [32], and the expression of VEGF-A is associated with poor prognosis [33], [34] and the presence of lymph node metastasis [35], raising the possibility that the biological roles of FLJ10540 and VEGF-A might be functionally linked in lung adenocarcinoma.

Given the major role of VEGF-A in regulating migration and invasiveness, we were first interested in whether VEGF-A could modulate FLJ10540 protein expression. The results showed that the protein expression level of FLJ10540 was upregulated in a VEGF-A dose-dependent manner in CL_1-0_ lung cancer cells (Figure 4B, left panel). We next asked whether the antagonistic effect of SU5416, a VEGFR-2 tyrosine kinase inhibitor, on biological activities of VEGF-A could be attributed to the inhibition of FLJ10540 protein overexpression induced by VEGF-A in lung cancer cells. The results demonstrated that upregulation of FLJ10540 in the presence of VEGF-A was inhibited by simultaneous addition of SU5416 in a dose-dependent manner (Figure 4B, middle panel). On the contrary, the protein level of FLJ10540 was not affected by treatment with VEGF-B (Figure 4B, right panel). In fact, *FLJ10540* does not share similar expression patterns with *VEGF-B*, at least not in our lung cancer microarray, supporting the idea that synexpression patterns could be used to infer the function of unknown genes [27].

### VEGF-A-induced FLJ10540 overexpression and translocation enhances lung cancer cell migration and invasion through an EMT-independent pathway

To test the biological significance of VEGF-A-induced upregulation of FLJ10540, we examined the influence of FLJ10540 on migration and invasion in the presence or absence of VEGF-A. First, we examined whether parental CL_1-0_ cells could display cell motility after stimulation by VEGF-A. In figure 4C, CL_1-0_ cells exhibited more migration and invasive abilities in the presence of VEGF-A alone than VEGF-A combined with SU5416 or vehicle alone, suggesting that the addition of VEGF-A significantly increased the migration and invasion of CL_1-0_ cells, which was paralleled by an increase in FLJ10540 levels. Similar results were found in FLJ10540 CL_1-0_-stable transfectants, with or without VEGF-A stimulation (Figure 4D).

To test whether the subcellular localization of FLJ10540 could be altered in lung cancer cells upon VEGF-A stimulation, we adopted the indirect immunofluorescence approach to observe FLJ10540 localization in CL_1-0_ lung cancer cells either with or without VEGF-A stimulation. As shown in figure 4E, VEGF-A treatment not only resulted in an increase in FLJ10540 protein expression, but it also promoted the translocation of a fraction of FLJ10540 to the cell membrane. In addition, the differences in cell morphology were more apparent, highlighting the extension of VEGF-A treated cells (Figure 4E, 30 min and 180 min) as compared with the rounded CL_1-0_ cells (Figure 4E, 0 min). These morphological changes prompted us to examine the phenomenon of epithelial-mesenchymal transition (EMT). During tumorigenesis, EMT may increase the motility and invasiveness of cancer cells, and malignant transformation may be associated with signaling pathways promoting EMT [36]. A previous study also indicated that extracellular signals induced by VEGF-A are strongly implicated in EMT in human pancreatic carcinoma cells [37]. To examine whether FLJ10540 might mediate VEGF-A-induced EMT, thereby enhancing cell migration and invasion, we used H1299 and CL_1-0_ lung cancer cells, with or without VEGF-A stimulation, to address this question. However, despite increasing cell motility by overexpressing FLJ10540 or decreasing cell motility by FLJ10540-mediated siRNAs, differences in FLJ10540 levels did not in any way alter the expression levels of any of the marker proteins associated with EMT, such as E-cadherin and vimentin (data not shown). Therefore, FLJ10540 seems to mediate migration and invasion effects of VEGF-A independently of EMT and does not appear to play a role in the regulation of EMT, at least in the lung cancer cell lines we analyzed.

### A positive correlation in protein expression of FLJ10540/VEGF-A and FLJ10540/AKT-p is observed in clinical specimens of lung adenocarcinoma at variable stages

**Figure 5:**
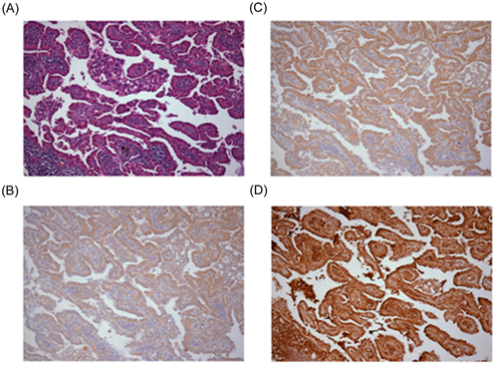
Histopathologic and immunophenotypic findings in lung adenocarcinomas with immunoreactivity for VEGF-A, FLJ10540, and phosphorylated AKT. (A) The tumor has a papillary growth pattern (Hematoxylin & Eosin, 100×). (B) The tumor cells are immunoreactive with VEGF-A, with a staining intensity score of 2 (100×). (C) The tumor cells are immunoreactive with FLJ10540, with a staining intensity score of 2 (100×). (D) The tumor cells are immunoreactive with phosphorylated AKT, with a staining intensity score of 3 (100×).

VEGF signals are partially tramitted through a PI3K-mediated signaling cascade, which includes AKT [38], and FLJ10540 could form a complex with PI3K in HCC [13]. These observations raise the possibility that VEGF-A/FLJ10540/PI3K/AKT might be in the same pathway, despite the fact that the gene expression patterns of *AKT* in our lung cancer microarray dataset did not exhibit any correlation with *FLJ10540*. This is not surprising, because the activation state of the PI3K signaling pathway is monitored by phosphorylated AKT. Therefore, we set up a tissue microarray, containing 273 archived lung adenocarcinomas at variable stages, to examine the protein expression patterns among these molecules. The immunohistochemical studies of HE staining (Figure 5A), VEGF-A (Figure 5B), FLJ10540 (Figure 5C), and phosphorylated AKT (Figure 5D) were performed on these lung adenocarcinoma specimens. The correlation between each paired IHC score of AKT-p, VEGF, and FLJ10540 was analyzed by the Spearman's rank tests. The results showed that there were positive correlations between VEGF and FLJ10540 (correlation coefficient = 0.36, p<0.001) and AKT-p and FLJ10540 (correlation coefficient = 0.22, p = 0.001). These results seem to imply that FLJ10540 may participate in a VEGF-A-mediated PI3K/AKT pathway in lung adenocarcinoma.

### Activation of PI3K/AKT is critical for FLJ10540-induced lung cancer cell migration and invasion upon VEGF-A stimulating

**Figure 6:**
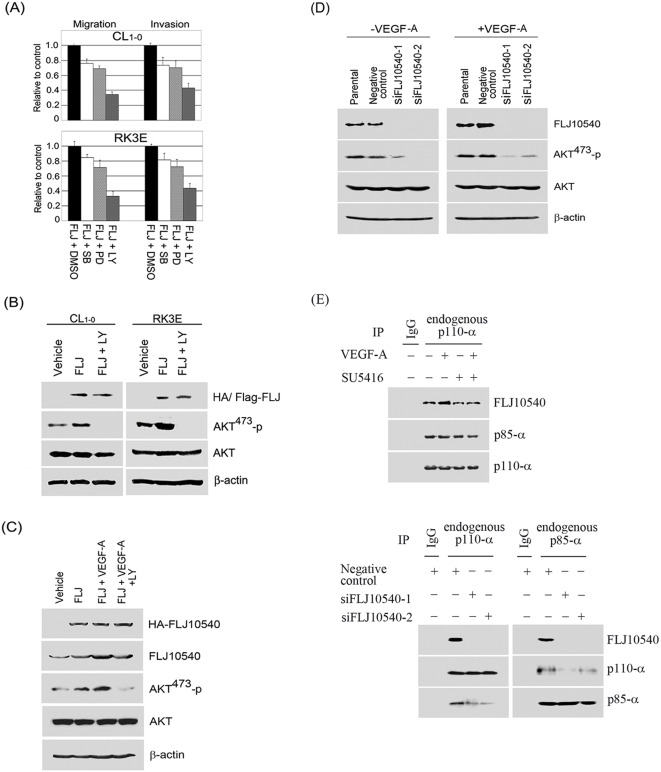
FLJ10540 alone or increased FLJ10540, mediated by VEGF-A stimulation, not only modulates cell migration and invasion through the PI3K/AKT signaling pathway, but also reinforces PI3K complex formation. (A) Vehicle-CL_1-0_, CL_1-0_-FLJ10540, vehicle-RK3E, and RK3E-FLJ10540 infected cells were serum-starved for 24 hours and treated with or without the indicated inhibitors, including SB202190, PD98059, and LY294002 for 2 hours. The migration and invasion ratios of vehicle-CL_1-0_, CL_1-0_-HA-FLJ10540, vehicle-RK3E, and RK3E-Flag-FLJ10540 infected cells were determined as previously described. (B) CL_1-0_ and RK3E-expressing FLJ10540 cells were treated with or without LY294002 at the final concentration of 10 µM. The total cell lysates were subjected to immunoblot analysis for the unphosphorylated or phosphorylated form of AKT. β-actin was used as an internal loading control. (C) CL_1-0_ cells expressing HA-FLJ10540 were pre-treated with or without VEGF-A (20 ng/ml). After 10 min, LY294002 (10 µM) was added and cells were further incubated for 2 hours. The total cell lysates were subjected to immunoblot analysis for HA, FLJ10540, or the unphosphorylated or phosphorylated forms of AKT. (D) A negative control siRNA and two different FLJ10540 siRNAs (siFLJ10540-1 and siFLJ10540-2) were transfected into CL_1-0_ cells for 24 hours. After transfection, cells were treated with or without VEGF-A (20 ng/ml) for 10 min. Western blotting was performed as in (C). (E upper panel) Serum-starved CL_1-0_ cells were pre-treated with or without SU5416 for 2 hours; cells were then stimulated with or without 20 ng/ml VEGF-A for 10 min. Cell lysates were immunoprecipitated with polyclonal antibodies against p110-α or protein IgG (as a control), which was followed by immunoblotting with p110-α, p85-α, and FLJ10540. (E lower panel) A negative control siRNA and two different FLJ10540 siRNAs (siFLJ10540-1 and siFLJ10540-2) were transfected into CL_1-0_ cells for 24 hours. After transfection, cell lysates were immunoprecipitated with polyclonal antibodies against p110-α, p85-α, or protein IgG (as a control), followed by immunoblotting with p110-α, p85-α, and FLJ10540.

Tumor cells possess a broad spectrum of migration and invasion mechanisms that are associated with enhanced tumor metastasis [4]. To explore how many signaling transduction pathways might participate in FLJ10540-mediated cell migration and invasion in lung cancer cells, we first investigated three well-defined signaling pathways, namely the PI3K/AKT, p38, and MAPK pathways, using pharmacological inhibitors. Figure 6A and Figure S3A show that FLJ10540-CL_1-0_ and FLJ10540-RK3E expressing cells had increased levels of cell migration and invasion, which were strongly decreased by incubation with LY294002, and to a much lesser extent, with SB202190 and PD98059. The same was found when PI3K was inhibited with wortmannin, a PI3K inhibitor (data not shown). Moreover, elevated AKT-Ser^473^ phosphorylation was observed in CL_1-0_-FLJ10540 and RK3E-FLJ10540 cells, compared with vehicle control cells, whereas FLJ10540-induced AKT-Ser^473^ phosphorylation was completely abolished by LY294002 (Figure 6B). These results indicate that FLJ10540-induced cell migration and invasion is mediated by the PI3K/AKT pathway.

We next investigated whether FLJ10540 might serve as a critical mediator of VEGF-A-induced cell migration and invasion. As Figure S3B shows, the FLJ10540-CL_1-0_ expressing cells, either with or without VEGF-A stimulation, had higher rates of cell migration and invasion, which were also strongly decreased by incubation with LY294002, but not by SB202190 and PD98059. Next, we took two approaches to provide additional evidence for the participation of AKT in FLJ10540-elicited cell migration and invasion upon VEGF-A treatment. First, we analyzed whether the increasing level of endogenous AKT-Ser^473^ phosphorylation could be observed in CL_1-0_-FLJ10540 transfected cells upon VEGF-A stimulation. Our data indicated that elevated AKT phosphorylation was observed in CL_1-0_-FLJ10540 cells upon VEGF-A stimulation, compared with the vehicle treated cells (Figure 6C). Furthermore, FLJ10540-induced AKT-Ser^473^ phosphorylation was completely abolished by LY294002 upon VEGF-A stimulation (Figure 6C). Our second goal was to examine if specific FLJ10540 siRNAs could affect endogenous AKT activation in CL_1-0_ cells, with or without VEGF-A stimulation. The data indicated that without VEGF-A stimulation of FLJ10540-depleted cells, the activation of AKT was greatly reduced compared to that seen in negative control cells (Figure 6D, left panel). Compared with negative control cells, the phosphorylated AKT levels were slightly increased upon VEGF-A stimulation in FLJ10540-depleted cells (Figure 6D, right panel), probably due to low residual FLJ10540 present in cells. Taken together, these results indicated that FLJ10540 is required for proper VEGF-A-dependent signaling and contributes to the PI3K/AKT pathway.

### VEGF-A-elicited FLJ0540 expression enhances the physical association with the PI3K complex

It has been observed that upon VEGF-A stimulation, increased levels of FLJ10540 can enhance AKT phosphorylation and that FLJ10540 can form a complex with PI3K [13]. This raises the possibility that increased levels of FLJ10540 could reinforce the association between p110-α and p85-α. To test this hypothesis, VEGF-A was used to treat CL_1-0_ lung cancer cells, either with or without SU5416. Cell lysates were immunoprecipitated with anti-p110-α antibody, and immuno-precipitates were analyzed by immunoblotting with anti-FLJ10540 and anti-p85-α. Cells with increased FLJ10540 levels, due to VEGF-A stimulation, had a slightly enhanced amount of the FLJ10540/p110-α/p85-α complex, as compared with cells without VEGF-A treatment (Figure 6E, upper panel). However, the affinity of the FLJ10540/p110-α/p85-α complex was decreased when cells were treated with SU5416 alone or with SU5416 combined with VEGF-A. To further confirm the association between the FLJ10540-PI3K complexes, we used FLJ10540-mediated siRNAs to address this question. Our data indicated that the degree of physical association between p110-α and p85-α was significantly decreased when endogenous FLJ10540 was depleted in CL_1-0_ lung cancer cells (Figure 6E, lower panel). These data indicated that increased FLJ10540, due to VEGF-A stimulation, may act as an important scaffold protein that can enhance and stabilize the PI3K complex in mammalian cells.

## Discussion

Identification of key regulators from microarray datasets remains a daunting task. Data integration and various bioinformatics approaches may substantially facilitate unmasking of a cryptic microarray dataset to identify targets and to dissect underlying mechanisms. Here, we show that FLJ10540 is the molecule interface between VEGF-A and the PI3K/AKT pathway. PI3K/AKT signal transduction has been shown to be a crucial element leading to survival, proliferation, migration, and invasion of human cells induced by VEGF-A [39]. Several reports have shown that the VEGF-A/VEGFR-mediated PI3K/AKT pathway is associated with signaling intermediates, such as phospholipase C-γ, GTPase-activating protein, Grb2-Gab1, and Shp2-Gab1 [29], [40], [41]. These interactions, including several PI3K-interacting partners, result in tyrosine phosphorylation of some of the potential substrates. However, the functional significance between PI3K-associated proteins and PI3K/AKT activation in VEGF-A signaling in lung adenocarcinomas has not been defined clearly. Here, our results show for the first time that FLJ10540 not only forms and stabilizes the PI3K complex upon VEGF-A stimulation, but also contributes to cell migration and invasion following PI3K/AKT activation in lung cancer cells. Our findings unravel a long unanswered question, finally demonstrating that FLJ10540 is the missing factor that bridges the activation mechanism of PI3K/AKT upon VEGF-A stimulation in cancer cells. Moreover, further studies are warranted to investigate whether FLJ10540, translocated from the cytoplasm to the plasma membrane after stimulation by VEGF-A, could interact with VEGFR or VEGFR-associated proteins directly or indirectly, prompting PI3K/AKT activation or participating in other pathways.

Various evidence points to the importance of deregulated PI3K/AKT signaling in lung cancer. Overexpression of both, the regulatory p85-α and the catalytic p110-α subunits of PI3K, was reported in primary human lung carcinomas [42], [43]. Genomic amplification of the *PIK3CA* (*p110-α*) gene was also documented in several lung cancers and in pre-invasive bronchial lesions, implicating the PI3K/AKT pathway in lung cancer development [44]. Several groups have reported that amplification, mutation, and high levels of phosphorylated AKT in tumor tissues from lung cancer patients correlated with poor prognosis in lung cancer patients in stage I of the disease or with primary tumors [42], [44], [45], [46], [47]. Moreover, in a panel of lung cancer cell lines, PI3K-dependent AKT phosphorylation and activation of PI3K also correlated with resistance to chemotherapy and radiotherapy [48], [49]. In short, inhibition of PI3K/AKT activation and disruption of the associated proteins that enhance PI3K complex formation have been widely proposed to represent promising therapeutic approaches for treatment of lung cancer.

In summary, the explosion in microarray studies has promised to shed light on potential disease markers and drug targets. This study illustrates that FLJ10540 is highly expressed in human lung adenocarcinomas, as well as in invasive lung cancer cell lines. This abnormal expression of FLJ10540 in mammalian cells is able to enhance cell migration and invasion. Moreover, the quantity of FLJ10540 in cells could serve as a critical mediator of VEGF-A-dependent biological events through activation of the PI3K/AKT signaling pathway, resulting in the progression of lung adenocarcinoma. In short, FLJ10540 could represent a potential target for the development of novel therapies for this widespread type of cancer.

## Materials and Methods

### Patients and tumor samples

A total of 56 samples from our previous study [50] were used for microarray analysis. These include pairwise samples from 26 patients, who underwent surgery for lung cancer at the Taipei Veterans General Hospital, and 4 lung cancer cell lines CL_1-0_, CL_1-1_, CL_1-5_ [51] and CL_1-5_-F4 [52]. The data have been deposited in NCBIs Gene Expression Omnibus (GEO) and are accessible through GEO series accession number GSE7670. No patients had previously received any neoadjuvant treatment, e.g. chemotherapy, before the surgery. The study protocol had the approval of the ethics committee at Taipei Veterans General Hospital. All patients gave informed consents and signed the consent form individually. Study samples, including tumor and adjacent normal tissues, were obtained during diagnostic biopsy, and adjacent normal tissues were derived from neighboring site outside of the tumor. Both tumor and adjacent non-tumor tissues for subsequent Q-RT-PCR studies were confirmed by pathologists.

### Quantitative reverse-transcription polymerase chain reaction (Q-RT-PCR)

To confirm the microarray data for *FLJ10540*, 16-pairs tumor and adjacent non-tumor tissues of lung patients' samples were used to validate the mRNA expression level of *FLJ10540* by using Q-RT-PCR, which was performed using a Taq-Man probe (ABI). Data were represented as mean±s.d. To analyze the distribution of tumor and adjacent non-tumor parts we performed the Wilcoxon signed rank test between two groups for statistical analysis. A *P* value of less than 0.05 was significant. *GAPDH* (ABI) was used as an internal control for comparison and normalization the data. Assays were performed in triplicate using Applied Biosystems Model 7700 instruments.

### Preparation of retroviral stock and infection

Retroviral vector-derived plasmid (1 µg) was introduced into 10^6^ DF-1 cells by FuGENE 6 as described previously [53]. Viral supernatant produced from these cells were harvested. For viral infection, actively dividing RK3E/*tv-a*-expressing cells or control cells were seeded. The next day, cells grown in fresh growth medium were mixed with viral supernatant to a total volume of 2.5 ml for 60 mm-plate and incubated at 37°C in 5% CO_2_ incubator for one hour. Afterwards, fresh growth medium was then added to the cell/virus mixtures.

### Cell culture, transient transfection and establishment of stable clones

All cell culture related reagents were purchased from Gibco-BRL. MDCK (clone 3B5) and RK3E cells were grown in DMEM medium containing 10% heat-inactivated FBS, 100 unit/ml penicillin and streptomycin (Gibco-BRL). CL_1-0_, CL_1-5_ and H1299 cells were grown in RPMI medium containing 10% heat-inactivated FBS, 100 unit/ml penicillin and streptomycin (Gibco-BRL). Transient transfection of HA-tagged FLJ10540 to CL_1-0_ was performed with Lipofectamine™ (Gibco-BRL) according to the manufacturer's instructions, whereas RK3E cells were infected with adenoviral Flag-tagged FLJ10540. CL_1-0_ cells stably expressing constructs were selected with 800 µg/ml G418 (Calbiochem). Individual clone was picked up and analyzed for exogenous FLJ10540 expression by western blotting. Each selected stable clones was lysed with extraction buffer as described below. Two double-stranded synthetic RNA oligomers (Ambion) (5′-GGGAGAAATTGCACACTTA-3′ and 5′-GGACTTTTAGCAAAGATCT-3′), deduced from human FLJ10540, and one *Silencer* negative control #4611G siRNA (Ambion) were used for siRNA experiments.

### Preparation of cell extracts, immunoprecipitation and western blot analysis

Cell-free lysates were prepared as described earlier [54]. Briefly, cells were harvested, washed with PBS, and lysed in extraction buffer (20 mM PIPES, pH 7.2, 100 mM NaCl, 1 mM EDTA, 0.1% CHAPS, 10% sucrose, 1 mM dithiothreitol, 1 mM phenylmethylsulfonyl fluorid (PMSF) and 1 mM Na_3_VO_4_,). For immunoprecipitation, 1 mg of total cell lysate was incubated with antibodies against target proteins and Protein A/G-agarose beads (Oncogene Research Product) to immunoprecipitate the target protein. Antibody against FLJ10540 (Abnova), AKT, AKT^473^-p (Cell Signaling), Flag (Sigma), HA (3F10, Roche), p110-α (Santa Cruz), p85-α (Santa Cruz) and β-actin (Sigma) were incubated with the membrane at room temperature for 1 h. The resulting IgGs were detected by incubation with secondary antibodies conjugated to HRP, and developed using the Western Lighting reagent.

### MTT assay for cell growth

Vehicle-CL_1-0_, FLJ10540-CL_1-0_ stable clones, vehicle-negative control and siRNAs FLJ10540 transfected cells of CL_1-5_ or H1299 (5×10^3^ ) were seeded in 96-well plates in RPMI containing 10% FBS for 24 h followed by MTT assay to quantify the cell growth. Data were normalized against OD_570_ value on day 0 of each vehicle control.

### Migration and Invasion Assay

The migration and invasion assays of the CL_1-0_ stable clones or the RK3E infected cells were evaluated using a 24-well Transwell (8-µm pore size polycarbonate membrane, Costar) chambers. For the migration assay, 5×10^3^ CL_1-0_ stable clones were suspended in 400 µl of DMEM or RPMI containing 10% FBS and were seeded into the upper chamber, while 600 µl of DMEM or RPMI containing 10% FBS was added to the outer side of the chamber. After being cultured in a 37°C, 5% CO_2_/95% air environment and allowed to adhere for 12 hours and then incubated with or without VEGF-A (20 ng/ml) for 10 min, cells on the upper surface of the membrane were removed by a cotton tip applicator and migratory cells on the lower membrane surface were fixed by methanol and stained with Giemsa (Sigma). Cell migration values were determined by counting from three independent membranes and then normalized using vehicle cells to give a relative ratio. For invasion assay, 80 µg/ml of Matrigel (BD Bioscience) was added to the upper surface of the membrane and allowed for gelling at 37°C for overnight. Cells (1×10^4^) in 400 µl of DMEM containing 10% FBS were seeded to the upper chamber, and 600 µl of DMEM containing 10% FBS was added to the outer side of the chamber. The rest of procedures for invasion assay were the same as in the migration assay.

### Tissue Microarray Analysis

Tissue cores manually removed from 273 archived lung adenocarcinomas were inserted into paraffin blocks to generate tissue arrays. The population includes 153 male and 120 female patients, with variable pathological stages (stage IA, 45 patients; IB, 105; IIA, 5; IIB, 18; IIIA, 49; IIIB, 21; IV, 12). Sections 5 µm in thickness were obtained and mounted on positively charged slides for immunohistochemical studies.

### Immunohistochemistry

Immunohistochemical detection of protein expression is performed on formalin-fixed, paraffin-embedded tissue sections of lung adenocarcinoma tissue arrays with standard procedures. The tissue sections were incubated at 4°C overnight with primary antibodies for VEGF-A (1∶800 dilution, BioGenex, sc-152), FLJ10540 (1∶800 dilution) and AKT^473^-p (1∶100 dilution). The intensities of the immunohistochemical staining were scored based on the following criteria: no or minimal staining in <10% of the tumor cells, score 0; faint, barely visible staining in >10% of the tumor cells, score 1; weak to moderate staining in >10% of the tumor cells, score 2; strong staining in >10% of the tumor cells, score 3.

### Statistical analyses

The correlation between each paired IHC scores of AKT^473^-p, VEGF and FLJ10540 were analyzed by Spearman's rank tests. A p value <0.05 was considered statistically significant. All analyses were carried out using statistics software SPSS 12.0 edition.

### Immunofluorescence microscopy

CL_1-0_ cells were incubated with VEGF (20 ng/ml) for 30 min or 180 min, the cells were washed with PBS and fixed with 3.7% formaldehyde in PBS for 15 min at 25°C. Cells on the coverslips were blocked with 5% BSA (Sigma) in PBS. The fixed cells were probed with anti-FLJ10540 antibody (1∶400) for 12 hrs at 4°C and then washed three times with 0.1% Triton X-100 in PBS. Immunofluorescent cell images were acquired using an Olympus Fluoview FV1000 Confocal laser scanning microscope (Olympus). Images were processed with Adobe Photoshop software (Adobe Systems). All data were collected during the same session to avoid potential differences due to fading.

## Supporting Information

Figure S1 Oncology. Molecular portraits of lung adenocarcinoma. (A) Supervised hierarchical clustering showed the 826 (634 down- and 192 up-regulated) transcript expression patterns of 26-pairwise lung adenocarcinoma samples. The results were shown in a dendrogram format, in which rows represent individual transcripts and columns represent tissue samples. Especially, FLJ10540 was shown in one of 192 up-regulated transcripts. The color in each cell reflected the expression level of the corresponding tissue, relative to its median expression level. The scale extends from fluorescence ratios of 0.25 to 4 relative to the median level for all samples. (B) The mRNA expression level of FLJ10540 was determined by Q-RT-PCR in 16 lung cancer patients. Overexpression of FLJ10540 was observed in 15 out of 16 lung cancer patient samples. The results were normalized against the expression level of GAPDH mRNA in each sample.
(8.38 MB TIF)

Figure S2 Oncology. FLJ10540 overexpression enhances motility of RK3E cells. (A) FLJ10540 stable clones of retrovirus infected Flag-tagged FLJ10540 in RK3E cells was established. The cell lysates were subjected to immunoblot analysis with anti-Flag antibody. (B) For the migration assay, 5×103 cells of vehicle-RK3E and RK3E-FLJ10540 infected cells were seeded into the top of a Transwell insert. After 24 hours, the cells on the topside were scraped, and the cells that migrated to the bottom were fixed and stained with Giemsa. The migration photography results of vehicle-RK3E and RK3E-FLJ10540 infected cells are shown (200×). The migration relative-folds of vehicle-RK3E and RK3E-FLJ10540 infected cells were normalized with vehicle control and presented diagrammatically. (C) For the invasion assay, 1×104 cells were seeded after Matrigel was added. The invasion photography results of vehicle-RK3E and RK3E-FLJ10540 infected cells are shown (200×). The invasion relative-folds of stable clone and infected cells were normalized against vehicle cells and represented diagrammatically. All of the data represent the mean±s.d. of three independent experiments.
(9.12 MB TIF)

Figure S3 Oncology. FLJ10540 mediates cell migration and invasion through the PI3K/AKT signaling pathway. (A). The migration and invasion photography results of vehicle-CL1-0, CL1-0-FLJ10540 stable clones, vehicle-RK3E and RK3E-FLJ10540 infected cells were treated with or without LY294002 at the final concentration of 10 mM are shown (200×). The data represent the mean±s.d. of three independent experiments. (B) Vehicle-CL1-0 and CL1-0-FLJ10540 stable clones on the Transwell insert were serum-starved for 24 hours and treated with or without the indicated inhibitors for 2 hours. Cells were then stimulated with or without VEGF-A at the final concentration at 20 ng/ml for 10 min. The migration and invasion ratios of vehicle-CL1-0 and CL1-0-FLJ10540 stable clones were determined. The data represent the mean±s.d. of three independent experiments.
(8.56 MB TIF)
