# Supplementary figures and images for: VEGFA Upregulates FLJ10540 and Modulates Migration and Invasion of Lung Cancer via PI3K/AKT Pathway
Source: PLoS One. 2009 Apr 1;4(4):e5052. doi: 10.1371/journal.pone.0005052 (PMC2659802; doi:10.1371/journal.pone.0005052)

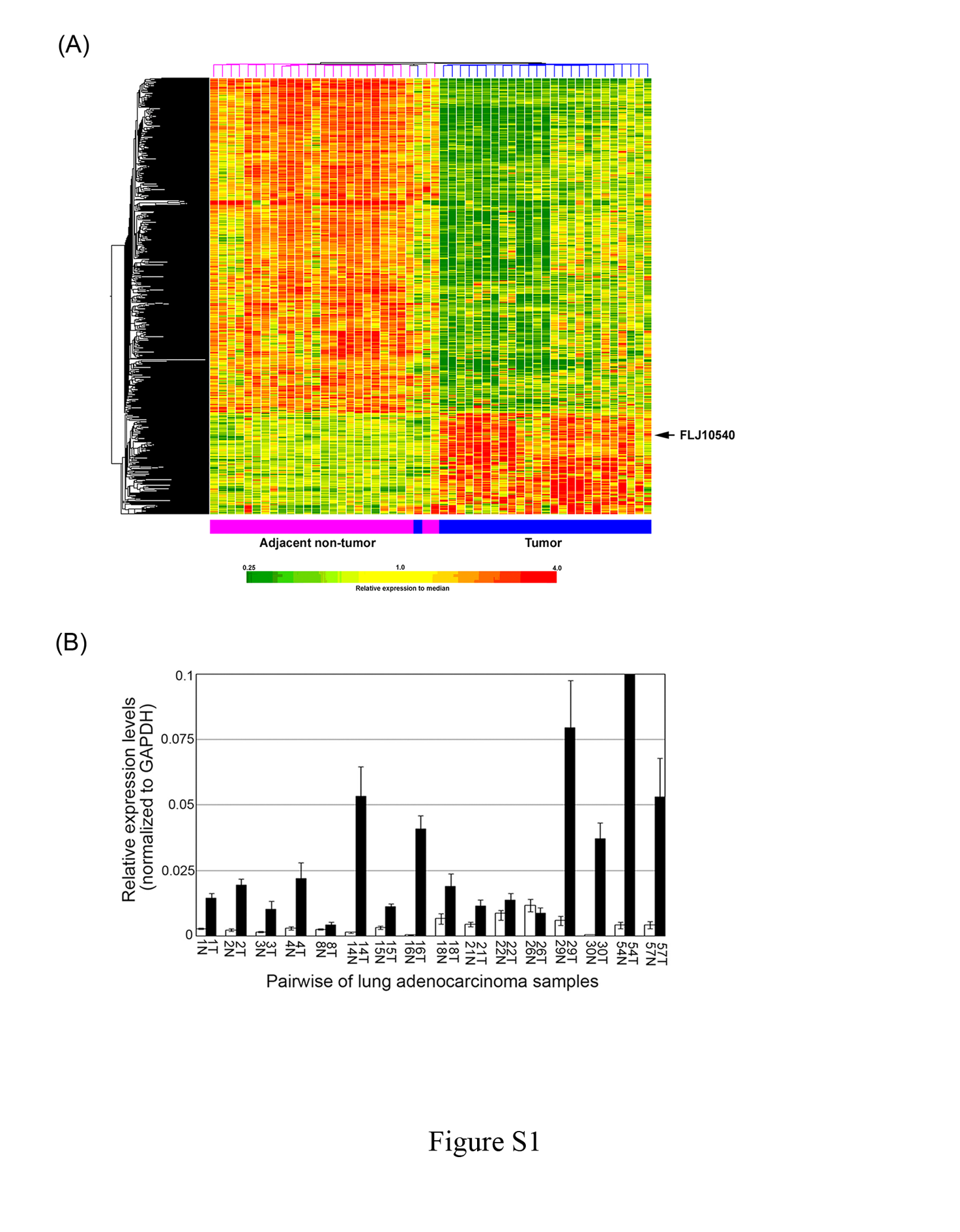

Supplement: Figure S1 — Oncology. Molecular portraits of lung adenocarcinoma. (A) Supervised hierarchical clustering showed the 826 (634 down- and 192 up-regulated) transcript expression patterns of 26-pairwise lung adenocarcinoma samples. The results were shown in a dendrogram format, in which rows represent individual transcripts and columns represent tissue samples. Especially, FLJ10540 was shown in one of 192 up-regulated transcripts. The color in each cell reflected the expression level of the corresponding tissue, relative to its median expression level. The scale extends from fluorescence ratios of 0.25 to 4 relative to the median level for all samples. (B) The mRNA expression level of FLJ10540 was determined by Q-RT-PCR in 16 lung cancer patients. Overexpression of FLJ10540 was observed in 15 out of 16 lung cancer patient samples. The results were normalized against the expression level of GAPDH mRNA in each sample. (8.38 MB TIF) [file pone.0005052.s001.tif]

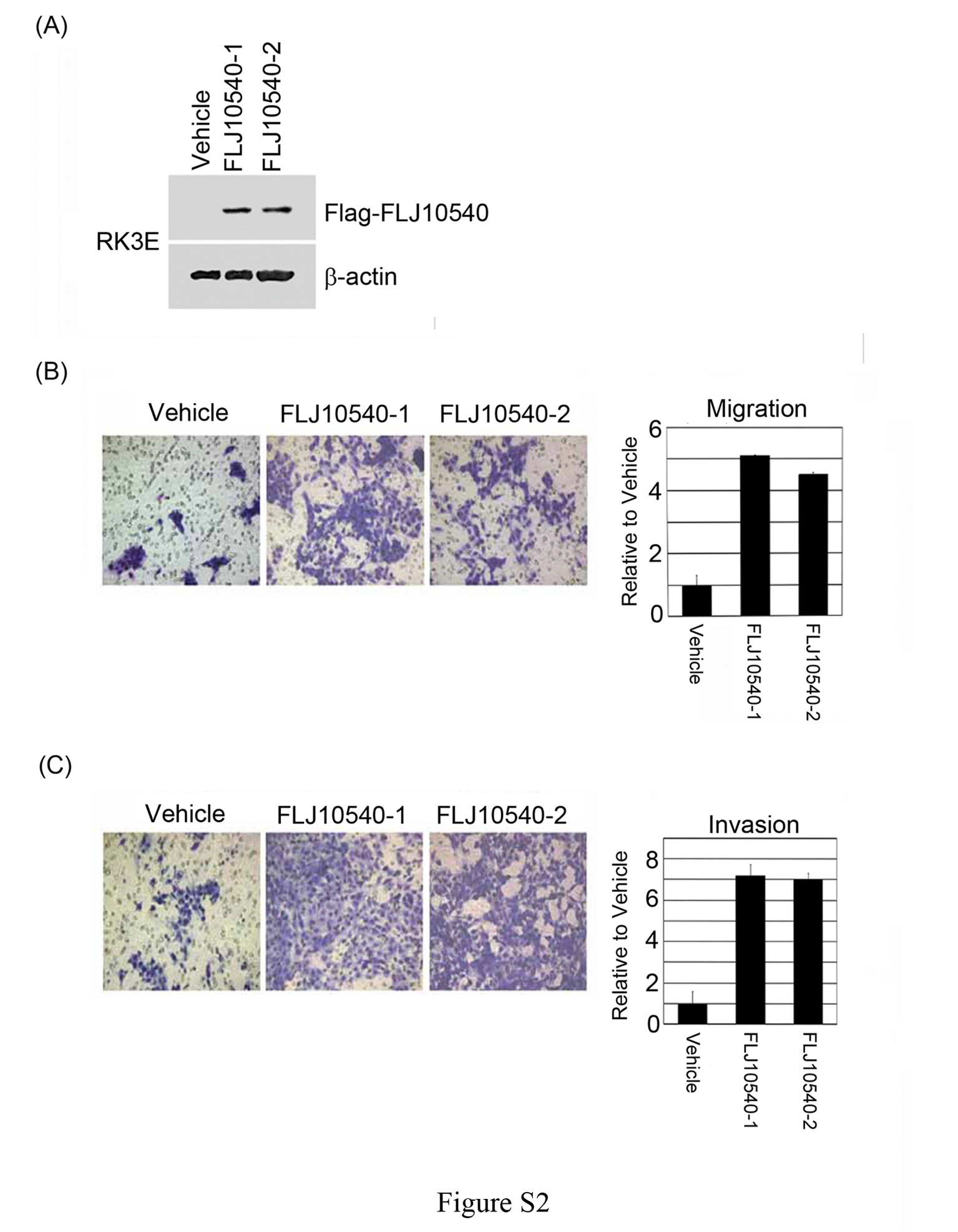

Supplement: Figure S2 — Oncology. FLJ10540 overexpression enhances motility of RK3E cells. (A) FLJ10540 stable clones of retrovirus infected Flag-tagged FLJ10540 in RK3E cells was established. The cell lysates were subjected to immunoblot analysis with anti-Flag antibody. (B) For the migration assay, 5×10^3^ cells of vehicle-RK3E and RK3E-FLJ10540 infected cells were seeded into the top of a Transwell insert. After 24 hours, the cells on the topside were scraped, and the cells that migrated to the bottom were fixed and stained with Giemsa. The migration photography results of vehicle-RK3E and RK3E-FLJ10540 infected cells are shown (200×). The migration relative-folds of vehicle-RK3E and RK3E-FLJ10540 infected cells were normalized with vehicle control and presented diagrammatically. (C) For the invasion assay, 1×10^4^ cells were seeded after Matrigel was added. The invasion photography results of vehicle-RK3E and RK3E-FLJ10540 infected cells are shown (200×). The invasion relative-folds of stable clone and infected cells were normalized against vehicle cells and represented diagrammatically. All of the data represent the mean±s.d. of three independent experiments. (9.12 MB TIF) [file pone.0005052.s002.tif]

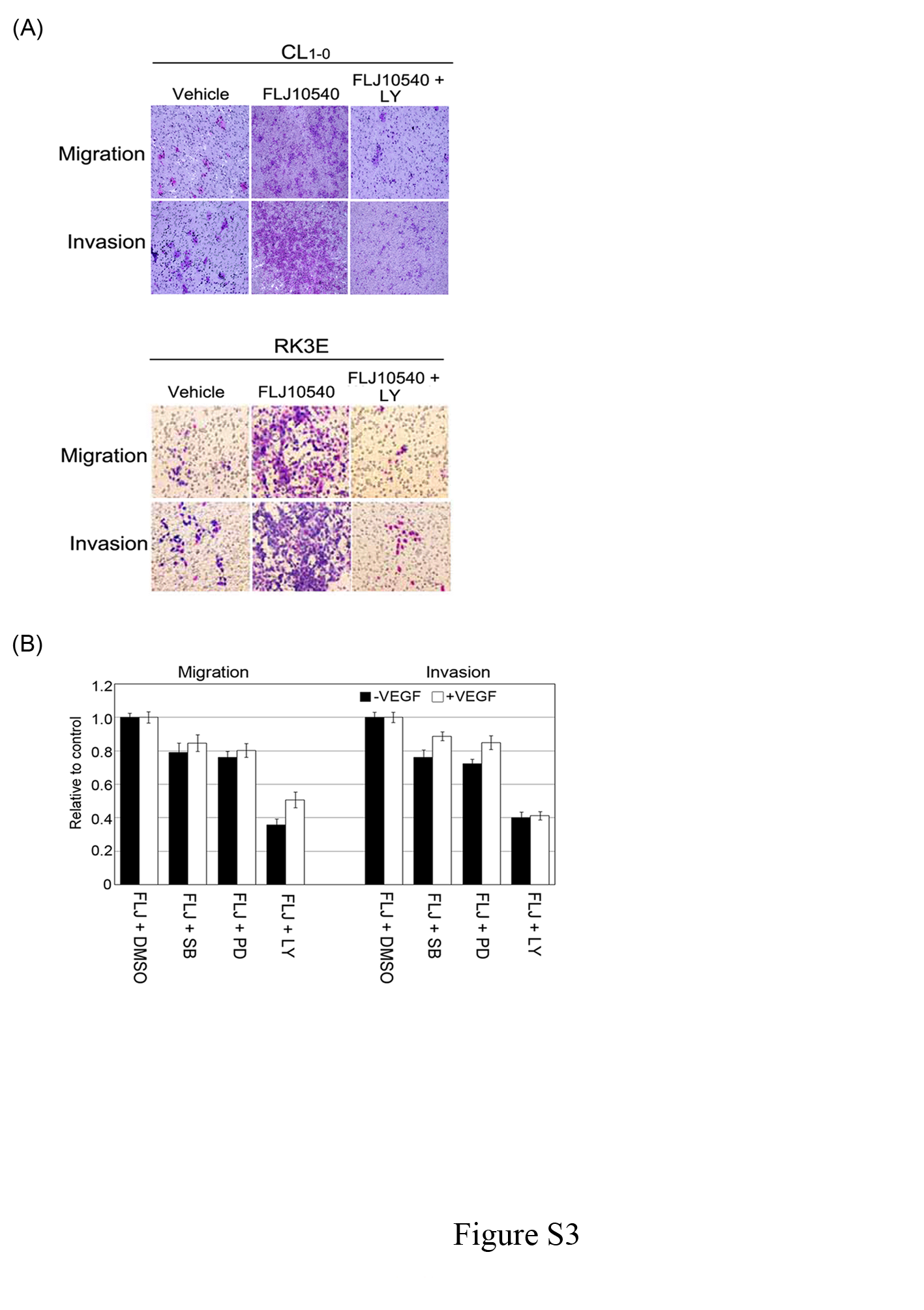

Supplement: Figure S3 — Oncology. FLJ10540 mediates cell migration and invasion through the PI3K/AKT signaling pathway. (A). The migration and invasion photography results of vehicle-CL1-0, CL1-0-FLJ10540 stable clones, vehicle-RK3E and RK3E-FLJ10540 infected cells were treated with or without LY294002 at the final concentration of 10 mM are shown (200×). The data represent the mean±s.d. of three independent experiments. (B) Vehicle-CL1-0 and CL1-0-FLJ10540 stable clones on the Transwell insert were serum-starved for 24 hours and treated with or without the indicated inhibitors for 2 hours. Cells were then stimulated with or without VEGF-A at the final concentration at 20 ng/ml for 10 min. The migration and invasion ratios of vehicle-CL1-0 and CL1-0-FLJ10540 stable clones were determined. The data represent the mean±s.d. of three independent experiments. (8.56 MB TIF) [file pone.0005052.s003.tif]
